# Safety and efficacy of single-dose preoperative intravenous dexamethasone on post-operative nausea and vomiting following breast surgery at Korle-Bu Teaching Hospital

**DOI:** 10.4314/gmj.v54i4.2

**Published:** 2020-12

**Authors:** Papa K G deGraft-Johnson, Robert Djagbletey, Henry K Baddoo, Ernest Aniteye, Raymond Essuman, George Aryee, Pokua Sarpong, Ebenezer O Darkwa

**Affiliations:** 1 Department of Anaesthesia, Korle-Bu Teaching Hospital, Accra, Ghana; 2 Department of Anaesthesia, University of Ghana Medical School, Korle-Bu, Accra, Ghana

**Keywords:** Dexamethasone, breast surgery, postoperative, nausea, vomiting

## Abstract

**Objectives:**

Dexamethasone has beneficial effects on postoperative nausea and vomiting, however, many clinicians have raised legitimate concerns regarding its effect on blood glucose concentrations. This study determined the safety and efficacy of a single pre-operative dose of dexamethasone for PONV prophylaxis in patients undergoing breast surgery.

**Design:**

Prospective, double-blind, placebo-controlled trial

**Setting:**

Surgical wards of the Korle-Bu Teaching Hospital, Accra, Ghana

**Participants:**

The study was conducted among breast surgery patients. They were consecutively recruited and randomized into two groups: dexamethasone (*n* = 47) and placebo (*n* = 47).

**Interventions:**

Patients in the dexamethasone group received 8mg (2mls of 4mg/ml) dexamethasone while those in the placebo group received 2mls of saline intravenously. PONV impact scores and blood glucose levels were recorded at 4, 8 and 24 hours postoperatively.

**Main outcome measures:**

Incidence of PONV and blood glucose levels

**Results:**

The incidence of postoperative nausea (PON) was lower in the dexamethasone group compared with the placebo group (12.8% vs. 29.8%; *p*-value= 0.044). There was no significant difference in the incidence of postoperative vomiting (POV) and PONV between the two groups. Blood glucose levels were higher in the dexamethasone group throughout the study period and significant at 8 and 24 hours postoperatively (*p* < 0.05). There was no difference in the incidence of clinically significant hyperglycemia between the groups (*p*-value = 0.169).

**Conclusion:**

A preoperative intravenous dexamethasone 8mg, reduces PON but not POV or PONV in breast surgery without clinically significant postoperative hyperglycemia.

**Funding:**

Non declared

## Introduction

Breast surgery ranges from minor procedures such as lumpectomies to major and complex procedures such as mastectomy with breast reconstruction. Breast cancer, the commonest female malignancy, is often the main reason for breast surgery at the Korle-Bu Teaching Hospital. Common postoperative morbidity associated with breast surgery includes postoperative pain, postoperative nausea and vomiting (PONV), seroma formation and chronic pain syndromes.^1^

The incidence of PONV following breast surgery is reported between 34% and 65%.^2^ Women undergoing mastectomy and axillary clearance have a higher risk of developing PONV, with a reported incidence of 60%–80% if prophylactic anti-emetics are not administered.^3^,^4^ Dexamethasone is the traditional drug of choice for the prophylaxis of postoperative nausea and vomiting due to its efficacy.^3^,^5^–^7^ The efficacy of dexamethasone in preventing nausea and vomiting has been shown to be at par with other anti-emetics like droperidol and ondansetron.^8^,^9^ Apfel *et al.*^9^, in their study, reported a 26% reduction in PONV among dexamethasone treated patients. Despite the beneficial effects of dexamethasone, many clinicians have raised legitimate concerns regarding the effect of dexamethasone on blood glucose concentrations and on the incidence of wound infections.^10^–^12^

A study done at Korle-Bu Teaching Hospital in adult patients undergoing various surgical procedures showed that over one-third of all postoperative patients experienced PONV of which only about 12% received treatment.^13^ The high cost of PONV prophylaxis using the recommended first-line agents (5-HT_3_ antagonists such as ondansetron), makes them unattractive in a resource-constrained environment such as Ghana, where cost of healthcare is a significant consideration for patients.

This study therefore aimed to determine the safety and efficacy of a single-dose preoperative intravenous dexamethasone on post-operative nausea and vomiting in patients undergoing breast surgery at the Korle-Bu Teaching Hospital.

## Methods

### Study site

This was a prospective randomized double-blind placebo-controlled trial carried out over an 18-month period (1^st^ July 2016 to 31^st^ December 2017) at the surgical block of the Korle-Bu Teaching Hospital, a major tertiary and the largest referral centre in Ghana. The hospital has a total bed capacity of approximately 1800 of which 120 are general surgical beds.

Elective breast surgeries undertaken include excision biopsies, microdochectomies, wide local excision with/without axillary lymph node dissection (ALND) and mastectomy with ALND.

### Ethical considerations

Approval for the study was obtained from the Korle-Bu Teaching Hospital Institutional Review Board (KBTHIRB), number: KBTH-IRB/00013/2016 and Trial registration number: PACTR201707002398224.

All patients who met the inclusion criteria and were recruited into the study were required to sign or thumbprint an informed consent form. Participants included in the study were assigned unique but confidential identifiers. Data extraction forms were securely kept, and electronic data was password protected. The keys and passwords required to access data were kept by the investigator who had the codes for the study.

### Study population

Patients with American Society of Anesthesiologists (ASA) classes I and II, aged between 18 and 70 years (inclusive) scheduled to have breast surgery at the Surgical Department during the study period were consecutively recruited.

Patients with known allergy to dexamethasone, history of gestational diabetes or diabetes mellitus, chronic steroid therapy, Immunosuppressed patients and patients on immunosuppressant drugs, history of motion sickness, PONV and patients with advanced breast disease for palliative procedures (e.g. toilet mastectomy) were excluded from the study. A total of 100 patients met the inclusion criteria and gave informed consent and were consecutively recruited into the study.

### Randomization and blinding

Recruited patients were subsequently randomized into two groups: (A) dexamethasone (*n* = 50) and (B) placebo (*n* = 50) using balloting without replacement. Investigators involved in the data collection and analysis, as well as the patients were blinded to the interventions. The code to the 2 groups was known only to one of the investigators (not involved in data collection and analysis) who only revealed the code after all the data had been collected and analysed.

### Sample size justification

The incidence of PONV in patients undergoing mastectomy with ALND who do not receive any PONV prophylaxis is reported between 60 to 80%.^4^ Gómez-Hernández *et al.*^14^ indicated that dexamethasone reduced PONV in patients undergoing breast cancer surgery by 40%. Assuming a mean incidence of PONV following breast surgery of 70% and a 40% reduction in PONV by dexamethasone, at the 95% confidence level and at a power of 80%, a sample size of 94 was adjudged to be adequate using the formula of Whitley *et al.*^15^

### Description of procedure

All patients who were recruited into the study had a pre-anesthetic review at the pre-anaesthetic clinic of Korle-Bu Teaching Hospital. The investigator who had the code to the identity of the groups prepared 2 ml syringes with the label ‘A’ or ‘B’ depending on the group a patient had been randomized to and handed it to the principal investigator. The syringes contained either 2mls of normal saline or 2mls of 4mg/ml (8mg) dexamethasone both colourless solutions.

All recruited patients had general anaesthesia and the blinded Anaesthetist administered the intervention (either 2ml of normal saline or 8mg dexamethasone) just before induction. Patients were induced with intravenous (IV) midazolam 1–2mg, IV fentanyl 1–2 µcg/kg and IV propofol 2–3mg/kg. Patients had endotracheal intubation and mechanical ventilation after muscle relaxation with IV vecuronium 0.1mg/kg. Anaesthesia was maintained using isoflurane in oxygen/air mixture. Intravenous fentanyl was used as part of the induction to blunt the pressor response to laryngoscopy and intubation as well as provide preemptive analgesia.

Due to its relatively short duration of action of approximately 30 minutes, intra-operatively, analgesia was maintained with IV morphine up to 0.1mg/kg and IV paracetamol 1g stat.

The PONV Impact Scale, described by Myles and Wengritzky^16^ was used to assess the PONV and Impact Scale Score recorded immediately on return to the recovery ward and at 4, 8 and 24 hours after the operation. The blood glucose concentrations were checked and recorded just before induction and at 4, 8 and 24 hours postoperatively. Blood glucose concentration was checked using OneTouchR SelectR glucometer (LifeScan Inc., USA). The pulp of a finger was cleaned with an alcohol-free cleaning solution prior to stabbing with a stylet to obtain a drop of blood for analysis. The number of patients who developed clinically significant postoperative hyperglycemia (defined as serum glucose concentration≥ 12mmol/l) was also recorded.

The postoperative pain management for all patients was 6 hourly administration of IV Paracetamol 1g and prn rescue opioid (IM Pethidine 1mg/kg) throughout the study period (i.e. the first 24 hours post-surgery). Patients who developed PONV were treated with intravenous (IV) metoclopramide 10mg stat which was repeated every 8 hours for the first 24 hours post-surgery.

### Data handling

Patient demographics, diagnosis and surgical procedure performed, incidence of nausea and vomiting, PONV Impact Score, Random blood glucose and any PONV treatment given were recorded on a data extraction form.

### Data entry and analysis

Source document verification was done to ensure accurate and credible data. Data of participants who after being recruited into the study showed study protocol violations were censored for removal from data analysis. Data collected was entered into a Microsoft Access database then exported into and analysed using SPSS version 20. Incidence was expressed as percentages. Categorical data was summarized as frequencies and proportions and continuous data as means ± standard deviation. Mean scores at various times were compared between treatment and control groups using repeated measures analysis of variance (ANOVA). Repeated measures ANOVA was also used to compare the blood glucose levels at the various time points between the treatment and control groups. Probability levels < 0.05 were considered statistically significant.

## Results

One hundred (100) participants were enrolled into the study from 1^st^ July 2016 till 31^st^ December 2017. Fifty (50) were randomized to the intervention group and 50 to the control group. Two (2) patients in the intervention group did not receive the allocated intervention because they were given steroids accidentally before the allocated intervention could be administered. Outcome data was incomplete for 1 patient in the intervention group.

**Figure 1:**
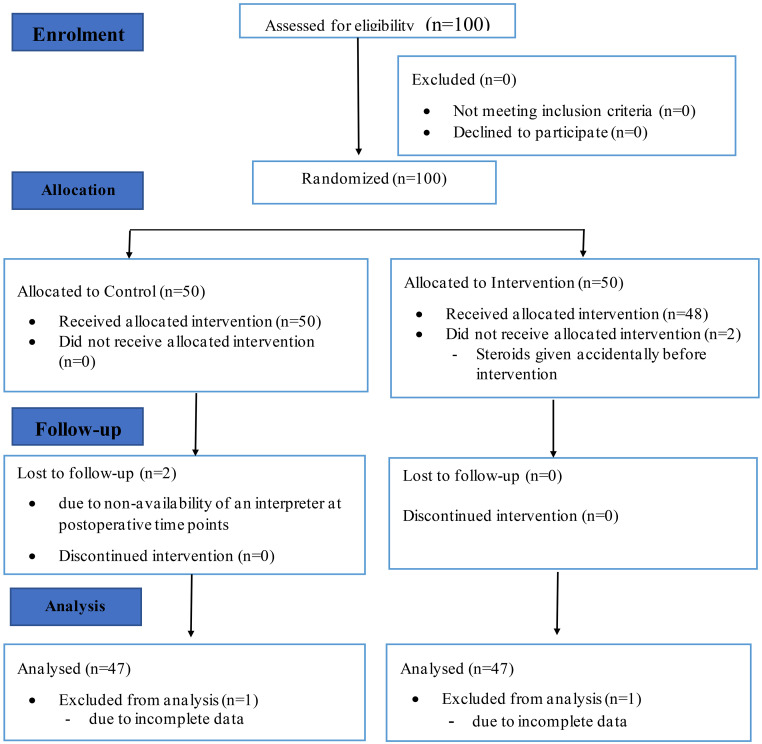
Consolidated Standards of Reporting Trials (CONSORT) diagram

In the control group, all 50 recruited patients received the allocated intervention. Two (2) patients in the control group were lost to follow-up because of a language barrier and the non-availability of an interpreter in the postoperative period. Outcome data was incomplete for 1 patient in the control group. There was complete data for 94 patients, 47 in each arm of the study (case to control ratio of 1:1) which was used in the data analysis as shown in the CONSORT diagram (Figure 1).

The age of the patients recruited into the study ranged from 21 to 70 years with a mean of 47.56 ± 11.68 years. Majority, 73 (84%) of the patients were aged between 31 and 60 years. Most patients, 91 (96.8%), recruited into the study were females. There was no significant difference in the demographic characteristics (age, weight, height and BMI), duration of surgery or anaesthesia, intra-operative and postoperative opioid used among the two groups (Table 1).

**Table 1:** Demographic and intra- and postoperative variables of patients' descriptive statistics in the two groups

Variable	Groups		t-statistic (df)	p-value
	Dexamethasone Mean ± std. dev.	Control Mean ± std. dev.		
**Age (years)**	49.55 ± 11.22	45.57 ± 11.91	1.67 (92)	0.099
**Weight (kg)**	78.43 ± 15.85	78.62 ± 17.67	-0.06(92)	0.956
**Height (m)**	1.62 ± 0.07	1.60 ± 0.06	1.57(92)	0.119
**BMI (kg/m^2^)**	29.81 ± 5.40	30.53 ± 6.62	-0.58(92)	0.566
**Duration of anesthesia (min)**	118.55 ± 38.91	122.94 ± 41.40	-0.53(92)	0.598
**Duration of surgery (min)**	87.55 ± 37.34	92.23 ± 34.43	-0.63(92)	0.529
**Intra-operative opioid**	4.68 ± 1.56	4.79 ± 1.23	-0.37 (92)	0.715
**Postoperative opioid**	113.75 ± 51.35	100.00 ± 60.93	0.93 (56)	0.358

There was no difference between the two groups in terms of administration of neo-adjuvant chemotherapy, indication for surgery and surgery performed as shown in Table 2. There was no significant difference in the incidence of postoperative vomiting between the two groups. The incidence of postoperative nausea was however, significantly lower in the dexamethasone group compared to the control group (12.8% versus 29.8%, *p*-value = 0.044) as shown in table 2.

**Table 2:** Diagnosis, preoperative chemotherapy, types of surgery incidence of PONV between groups

Variable		Groups		Chi-square/ Fisher's test	*p* value
		Dexamethasone	Control		
**Neo-adjuvant** **Chemotherapy**	Yes	15(31.9%)	15(31.9%)	0.00	1.000
	No	32(68.1%)	32(68.1%)		
**Diagnosis**	Breast ca	41(87.2%)	43(91.5%)	2.05	0.662
	Gynaecomastia	0(0.0%)	1(2.1%)		
	Duct ectasia	2(4.3%)	1(2.1%)		
	Others	4(8.5%)	2(4.3%)		
**Surgery**	WLE + Axillary clearance	18(38.3%)	13(27.7%)	6.24	0.136
	Mastectomy + Axillary clearance	20(42.6%)	31(66.0%)		
	Excision biopsy	3(6.4%)	1(2.1%)		
	Microdochectomy	1(2.1%)	0(0.0%)		
	Others	5(10.6%)	2(4.3%)		
**PONV**	Yes	5(10.6%)	9(19.1%)	1.34	0.247
	No	42(89.4%)	38(80.9%)		
**POV**	Yes	6(12.8%)	12(25.5%)	2.47	0.116
	No	41(87.2%)	35(74.5%)		
**PON**	Yes	6(12.8%)	14(29.8%)	4.07	0.044*
	No	41(87.2%)	33(70.2%)		

**Figure 2:**
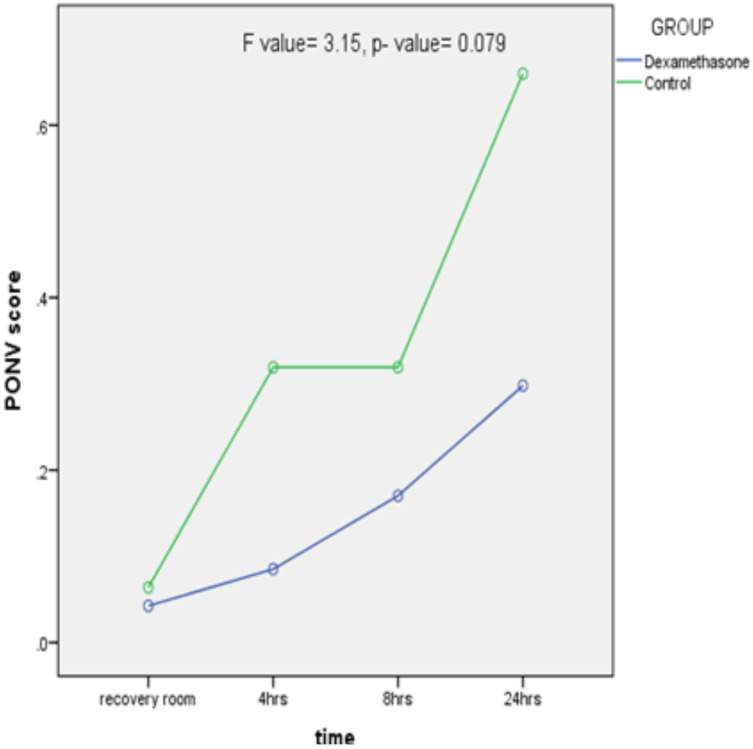
Postoperative nausea and vomiting (PONV) score over the 24-hour study period.

There was no significant difference in the incidence of PONV between the dexamethasone and control groups (10.6% versus 19.6%, *p* -value = 0.247). The mean PONV Impact Scale Score was lower in the dexamethasone group compared to the control during the study period. There was, however, no significant difference in clinically important PONV (PONV Impact Scale Score ≥ 5) between the two groups as shown in Figure 2.

## Discussion

The vast majority of patients (89.4%) in our study had breast cancer. Our study site is the largest referral hospital in Ghana and also the main center for referral of cases of breast malignancies in the country and may have influenced the significant proportion of the breast cancer cases observed.

The relatively higher cost of serotonin antagonists precludes their routine use as prophylaxis for PONV in low resource countries such as Ghana. On the other hand, a cheaper and equally effective^3^ alternative prophylaxis for PONV, dexamethasone, may be the preferred choice in such environments. Arsalani-Zadeh *et al.*^17^ have recommended the pre-emptive use of dexamethasone as part of a protocol aimed at enhancing recovery after breast surgery.

The low incidence of PONV among the controls (9.6%) in our study is in sharp contrast with that quoted in the literature (60%–80%).^4^,^5^,^18^ The populations in which PONV in breast surgery have been studied have mainly been Caucasian,^19^–^22^ Asian,^23^,^24^ or Hispanic^14^ in nature as compared to our study in a Ghanaian (African) population. Therefore, racial differences in the population studied may possibly have accounted for this difference.

Rodseth et al.^25^ in their study found that Africans had a highly significantly lower incidence of PONV than their non-African counterparts (27% vs. 45%, *p* <0.0001). Their study identified female sex, non-African ethnicity and the use of postoperative opioids to be independent predictors of PONV.^25^

Per the simplified Apfel PONV risk score,^22^ most of the patients in our study had at least 3 risk factors (female gender, non-smoker status and postoperative opioids) and will be considered at a high risk (60%) of PONV. Our overall incidence of PONV was however much lower (∼15%). Further studies are required to investigate effects of racial and genetic factors in the incidence and severity of PONV.

Although the incidence of PON, POV and PONV was relatively low in both groups in this study, those in the dexamethasone group had a significantly lower incidence of PON compared to the control group (*p*-value= 0.044) similar to findings of De Oliveira Jr *et al.*^27^. However, there was no significant difference in the incidence of POV (*p*-value= 0.116) between the two groups. Our study demonstrated that the administration of preoperative dexamethasone reduced the incidence of PONV by 45% when compared to placebo (*p*-value = 0.247) and this is similar to the findings by Gomez-Hernandez *et al*.^14^

A recent evidence-based review on risk assessment, prevention and treatment of nausea and vomiting after surgery under general anaesthesia, recommended prompt treatment of PONV, as the risk of it persisting or recurring is approximately 65%.^28^ Only 1 patient (in the control group) received rescue antiemetic (IV metoclopramide) throughout the study period despite the fact that a total of 14 patients (5 in the dexamethasone group and 9 in the control group) experienced PONV over the same period. The low treatment rate for PONV among these patients suggests that the management of PONV at the study site may still be suboptimal and remains a challenge over a decade after Amponsah^13^ estimated a treatment rate of approximately 1 in 10 patients.

The overall incidence of clinically important PONV in our study was only 2%, ten times lower than the value (20%) reported by Myles and Wengritzky.^16^ In their study, unlike ours, patients were pre-selected and only those identified to be experiencing PONV, receiving treatment for PONV or at risk of developing PONV were enrolled.

Thus, the method of patient selection and racial differences in the populations studied may account for the differences in findings.

In this study, the trend of blood glucose levels was similar in both groups, peaking between the 4^th^ and 8^th^ postoperative hours and declining thereafter and this is similar to the findings of Hans et al.^10^ The incidence of hyperglycemia in those receiving dexamethasone was not significantly different from controls (*p* = 0.169). In addition, there was a clinically unimportant increase in the peak postoperative glucose concentration of 1.54mmol/l (*p* <0.001) in the dexamethasone group similar to findings of Toner et al.^29^ Valid concerns raised about the effect of perioperative administration of steroids on postoperative morbidity include wound infection and hyperglycemia.^30^,^31^,^32^

No patient in our study had impaired wound healing or wound infection affirming the findings of Toner et al.^29^ that administration of perioperative glucocorticoids did not result in wound infections, impaired wound healing, anastomotic leak or operative site bleeding in patients undergoing non-cardiac surgery.

The administration of dextrose-containing fluids in the postoperative period may have also influenced the blood glucose levels measured. All cases of recorded hyperglycemia in this study responded favorably to reduction in the infusion rate of dextrose-containing fluid or a change to a non-dextrose containing fluid.

The incidence of PON was significantly lower among patients in the dexamethasone group compared to the placebo group. Though the incidence of POV and PONV were generally lower in the dexamethasone group, this was not found to be statistically significant.

Further studies are required to investigate effects of racial and genetic factors in the incidence and severity of PONV.

We caution generalization of findings of this study as it is a single-center study and may not reflect the national or African picture. A larger, multi-center trial is required to investigate the factors that influence the incidence of PONV in African patients.

## Conclusion

This study shows that a single-dose preoperative IV dexamethasone 8mg administered to patients (excluding diabetics and glucose-intolerant patients) undergoing breast surgery reduces postoperative PONV without significant adverse effects.

## Figures and Tables

**Figure 3:**
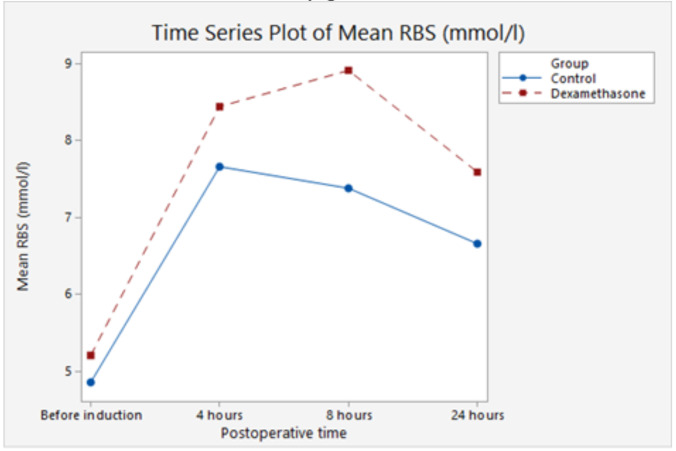
Changes in the blood glucose levels (RBS in mmol/l) over a 24-hour study period.
